# Intracellular peptides in SARS-CoV-2-infected patients

**DOI:** 10.1016/j.isci.2023.107542

**Published:** 2023-08-06

**Authors:** Luiz Felipe Martucci, Rosangela A.S. Eichler, Renée N.O. Silva, Tiago J. Costa, Rita C. Tostes, Geraldo F. Busatto, Marilia C.L. Seelaender, Alberto J.S. Duarte, Heraldo P. Souza, Emer S. Ferro

**Affiliations:** 1 Department of Pharmacology, Biomedical Sciences Institute, São Paulo 05508-000, Brazil; 2 Department of Pharmacology, Ribeirao Preto Medical School, Ribeirão Preto 14049-900, Brazil; 3 Department of Psichiatry, Medical School and Hospital das Clínicas, University of São Paulo, 01246-903 SP, Brazil; 4 Department of Surgery, Medical School and Hospital das Clínicas, University of São Paulo, 01246-903 SP, Brazil; 5 Department of Patology, Medical School and Hospital das Clínicas, University of São Paulo, 01246-903 SP, Brazil; 6 Department of Internal Medicine, Medical School and Hospital das Clínicas, University of São Paulo, 01246-903 SP, Brazil

**Keywords:** Virology, Public health, Microbiology

## Abstract

Intracellular peptides (InPeps) generated by the orchestrated action of the proteasome and intracellular peptidases have biological and pharmacological significance. Here, human plasma relative concentration of specific InPeps was compared between 175 patients infected with severe acute respiratory syndrome coronavirus 2 (SARS-CoV-2), and 45 SARS-CoV-2 non-infected patients; 2,466 unique peptides were identified, of which 67% were InPeps. The results revealed differences of a specific group of peptides in human plasma comparing non-infected individuals to patients infected by SARS-CoV-2, following the results of the semi-quantitative analyses by isotope-labeled electrospray mass spectrometry. The protein-protein interactions networks enriched pathways, drawn by genes encoding the proteins from which the peptides originated, revealed the presence of the coronavirus disease/COVID-19 network solely in the group of patients fatally infected by SARS-CoV-2. Thus, modulation of the relative plasma levels of specific InPeps could be employed as a predictive tool for disease outcome.

## Highlights

• SARS-CoV-2 infection leads to the modulation of specific InPeps levels in human plasma
• InPeps uncovered coronavirus-associated protein-protein interaction network
• The biological significance of InPeps was corroborated by the present study

## Graphical abstract

**Figure:**
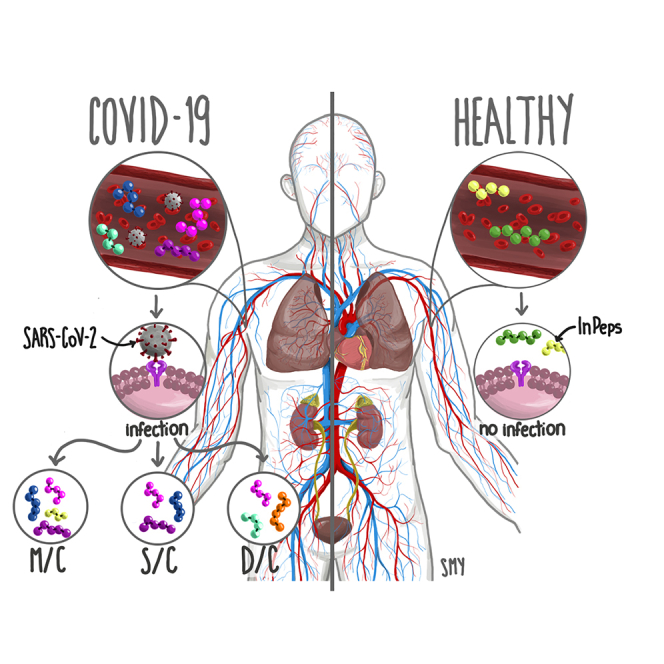

## Introduction

Circulating blood plasma connects the various compartments of the organism, promoting tissues/organs crosstalk, and warranting functional organization and accordance of adaptive responses. Human plasma peptide contents have been for decades associated with secreted, short-lived, neuronal and hormonal peptides, such as insulin, glucagon, angiotensin, and bradykinin. Recent advances in high-sensitive electrospray ionization mass spectrometry coupled with high performance nano liquid reversed-phase chromatography (ESI-nLC-MS/MS), allowed us to investigate proteome and peptidome from human fluids in far greater details.^1^ Perturbation of the human plasma proteome was shown to persist for up to 6 weeks following the first confirmed severe acute respiratory syndrome coronavirus 2 (SARS-CoV-2) infection, allowing to track symptoms of severity and antibody responses.^2^ Human urine peptidome in SARS-CoV-2-infected patients suggested that urinary peptide profiling generates candidate biomarkers even at early disease stages.^3^^,^^4^ It is noteworthy to mention that human urine and plasma have almost complete distinctive peptide profiles, suggesting that human plasma has a large pool of resident peptides, maintained by reabsorption in detriment of excretion during renal filtration.^4^^,^^5^^,^^6^ Despite the well-known function of bradykinin and angiotensin^7^^,^^8^ and fibrinogen-derived peptides,^9^^,^^10^^,^^11^^,^^12^^,^^13^^,^^14^^,^^15^ the biological function of most plasma resident peptides remains poorly investigated.

Considering that peptides play key roles in physiology as well as in several diseases, and are frequently employed for medication^16^^,^^17^^,^^18^^,^^19^^,^^20^ or diagnosis,^4^^,^^21^ the present report was designed to semi-quantitatively investigate, by using formaldehyde-derived isotope labeling, the human plasma peptidome in SARS-CoV-2-non-infected individuals and in SARS-CoV-2-infected patients. The results show variations in the relative levels of specific peptides according to the defined clinical status of the patients (i.e., non-infected vs*.* SARS-CoV-2-infected with moderate, severe, or fatal infection). Peptides identified in human plasma were 67% derived from intracellular, non-secreted proteins. Plasma peptides identified herein and elsewhere^6^^,^^22^ were compared with the collection of previously reported urinary peptides. Plasma and urinary peptidomes were distinctive in several biochemical aspects (i.e., precursor proteins, molecular weight, net charge, isoelectric point, hydrophobicity, aliphatic index, and instability index). The present results indicate that human plasma presents a large pool of resident peptides, whose relative levels vary according to the severity of SARS-CoV-2 infection. InPeps herein identified have unknown biological or pharmacological function, requiring further characterization.

## Results

Individuals diagnosed with SARS-CoV-2 infection (n = 175, 63 male, 100 female, and 12 undeclared sex) and non-infected (control) individuals (n = 45; 17 male and 28 female), were enrolled for this study (Tables 1 and S3). All SARS-CoV-2-infected patients enrolled in this study received supplemental oxygen, and according to the WHO guidelines^23^^,^^24^ could be considered as cases presenting the severe form of the COVID-19 disease. However, for the purpose of this plasma peptidome study, SARS-CoV-2-infected patients were sub-classified into: (1) patients not mechanically ventilated or with moderate infection (M); (2) patients mechanically ventilated or with severe infection (S); and (3) severe fatal infection that led to death during hospitalization (D). Non-SARS-CoV-2-infected individuals were considered “controls” (C).

**Table 1:** General clinical characteristics of patients investigated herein

	C	M	S	D
Age (years)	58.57 ± 19.59	59.24 ± 14.58	58.31 ± 12.79	66.86 ± 14.69
Sex (n)				
Male	17	22	41	22
Female	28	37	27	14
Not available	0	11	0	1

M, moderate infection; S, severe infection; D, infection that led to death during hospitalization; C, non-SARS-CoV-2-infected individuals Table S3 contains additional descriptions of patients’ clinical conditions, including pre-existence of obesity, diabetes, and/or hypertension, as well as hospitalization period (days), need of mechanical ventilation, and time in mechanical ventilation if needed (days).

**Figure 1:**
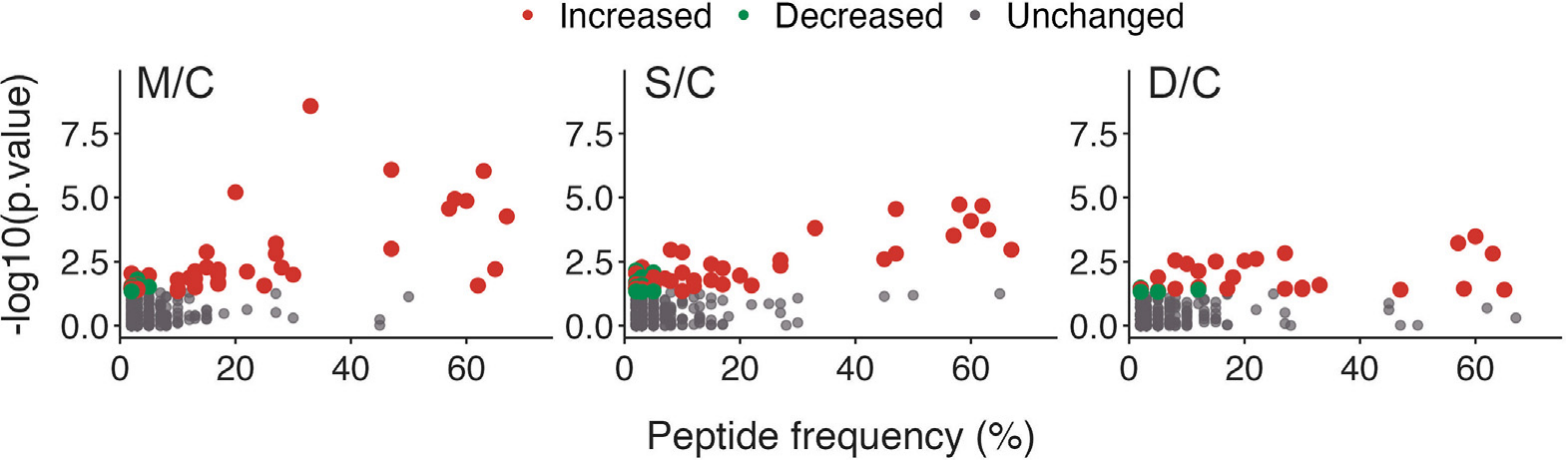
Plasma peptides frequency and their respective p value when comparing peptide ratios M/C, S/C, and D/C. Colored dots represent peptides whose relative ratio either remained unaltered (gray dots), significantly increased (red dots) or significantly decreased (green dots) [p value ≤ -log_10_ (5·10^−2^)]. See also Table S4.

Plasma peptidome analyses in this group of 220 individuals unveiled he presence of 2,466 distinct peptides of varied frequencies across mass spectrometry runs (Figure 1; Table S4). As the peptides were isotope-labeled their relative levels among groups M/C, S/C, or D/C could be compared. Several differentially regulated (increased or decreased) peptides were identified, with increased peptide ratios being more evident among SARS-CoV-2-infected patients (Figure 1).

Table 2 shows peptides with frequency ≥30% across mass spectrometry runs, and their semi-quantitatively evaluated ratios among groups MC, S/C or D/C. Peptides such as GIFTDQVLSVLKGEE, VESTSNSPSSS, IKERVPDSPSPAPSLEE, and GEGDFLAEGGGVR were identified in more than 50% of SARS-CoV-2-infected patients, and may thus present relevance for diagnosis; these more frequent peptides originated from nine proteins. Fibrinogen gave rise to four peptides, DACH1 yielded three peptides, and DACH2 generated two peptides (Table 2). The remaining proteins gave rise to only one peptide each (Table 2).

**Table 2:** Peptide frequency and group comparisons

	Freq. (%) ∗	M/C	S/C	D/C
**APOC2**				

GIFTDQVLSVLKGEE	60.00	↑	↑	↑

**ATP7A**				

VESTSNSPSSS	56.67	↑	↑	↑
DACH1				
IKERVPDSPSPAPSLEE	58.33	↑	↑	↑
KERVPDSPSPAPSLEE	33.33	↑	↑	↑
VPDSPSPAPSLEE	66.67	↑	↑	↔

**DACH2**				

ESPSPAPSLEE	45.00	↔	↔	↔
KERIPESPSPAPSLEE	46.67	↑	↑	↔

**FAT2**				

DGSDVSK	30.00	↔	↔	↑

**FIBA**				

ADSGEGDFLAEGGGVR	61.67	↑	↑	↔
DSGEGDFLAEGGGVR	65.00	↑	↔	↑
GEGDFLAEGGGVR	63.33	↑	↑	↑
SGEGDFLAEGGGVR	46.67	↑	↑	↑

**IRGQ**				

LLALPPASPSAARTKA	31.67	↑	↔	↑

**KNG1**				

RPPGFSPFR (bradykinin)	45.00	↔	↑	↔

**ZN541**				

SPSEESPPGPGG	50.00	↔	↔	↔

M, moderate infection; S, severe infection; D, infection that led to death during hospitalization; C, non-SARS-CoV-2-infected individuals. Freq. (%)∗, percentage of mass spectrometry runs whose peptide sequence was identified. Symbols indicate: ⟷, no change in peptide ratio, p > 0.05; ↑ or ↓, respectively, peptide ratio either increased or decreased, p < 0.05. See also Table S5 for results segmented by sex.

When categorized by sex, fewer peptides seem differentially modulated in patients infected by SARS-CoV-2, as compared to non-infected individuals (Table S5). Shortly, an increase in the peptide GIFTDQVLSVLKGEE occurred in females from groups M/C and S/C, and in males D/C. The peptide VESTSNSPSSS increased only in females, in M/C, S/C, and D/C. Peptide KERVPDSPSPAPSLEE increased in both males and females from groups M/C and S/C, but not in group D/C. The related peptide IKERVPDSPSPAPSLEE increased in both male and female from group M/C, and in males from group S/C, while the shorter peptide VPDSPSPAPSLEE increased only in females from the M/C group. Peptide KERIPESPSPAPSLEE increased in females from group M/C, whereas the peptide DGSDVSK was reduced in males from groups M/C and D/C. Fibrinogen peptide ADSGEGDFLAEGGGVR was increased in females from group S/C, the peptide SGEGDFLAEGGGVR was increased in males from M/C group, while the shorter peptide GEGDFLAEGGGVR appeared increased in males and females from M/C group.

**Figure 2:**
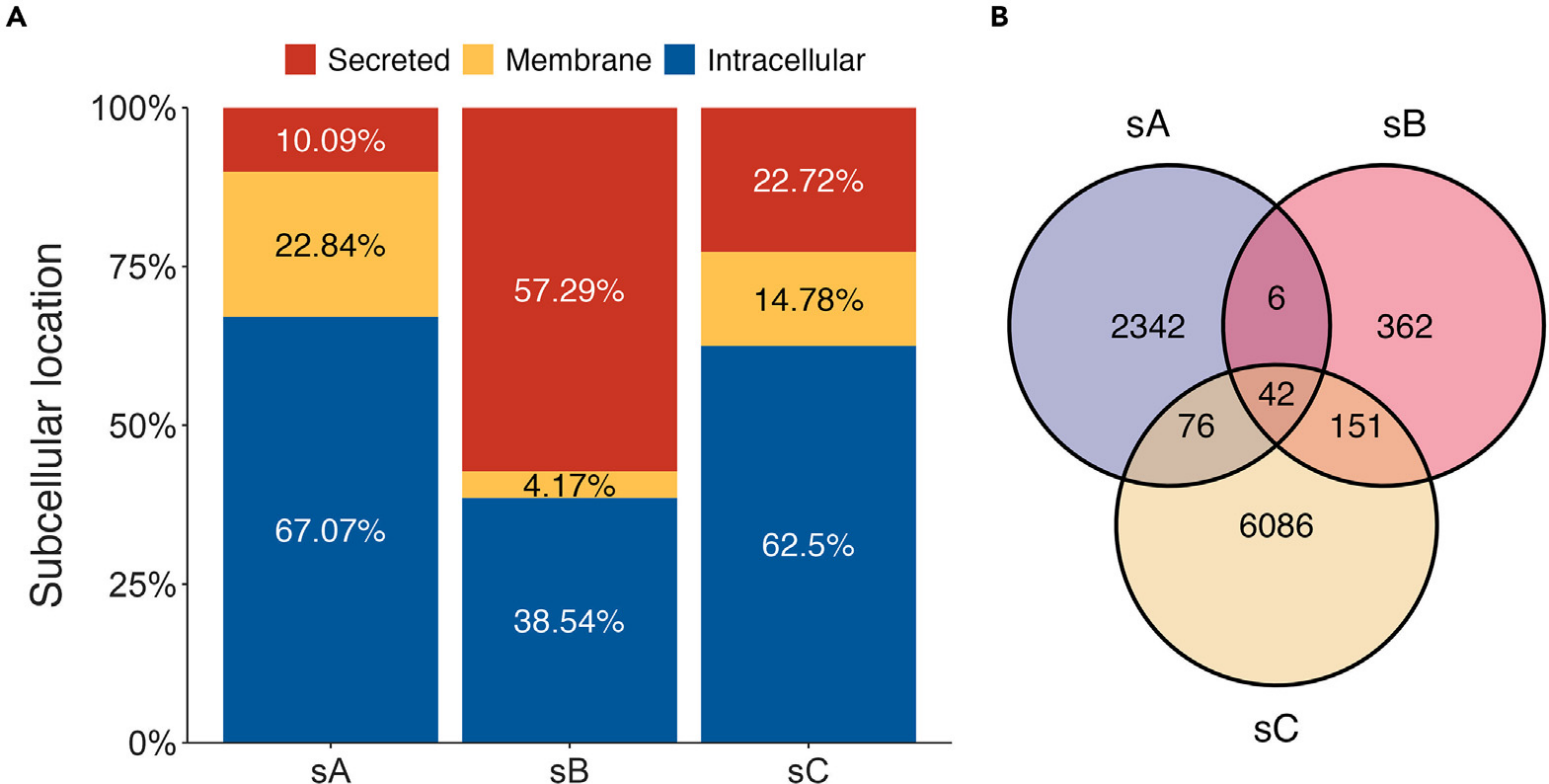
Comparative analyzes of human plasma peptidome studies (A) subcellular localization of proteins that originated the identified peptides. (B) Venn diagram of peptides identified across studies. sA, present report; sB, Magalhães et al*.*^5^ and *sC*, Parker et al.^25^ See also Table S6.

From all 2,466 unique peptides identified herein, 67.3% were considered InPeps as they originated from proteins whose predominant subcellular localization was either cytosolic, nuclear or mitochondrial (Figure 2A, sA); similar results were obtained considering only the most frequent peptides shown on Table 2. A smaller proportion of peptides identified in the human plasma were from proteins preferentially compartmentalized in the cellular membranes (22.72%; Figure 2A, sA), or devoted to the secretory pathway (9.91%; Figure 2A, sA).

Considering that one protein can originate more than one peptide, the percentage and the respective subcellular localization of the precursor proteins giving rise to unique peptides were then investigated. Secreted proteins were observed to give rise to the greatest number of plasma peptides per protein (13.60 peptides per precursor protein), while intracellular proteins generated 3.58 peptides per precursor protein, and membrane proteins generate 2.49 peptides per precursor protein.

Comparisons between the present study and previous plasma peptidomics studies^6^^,^^22^ revealed both similarities and discrepancies (Figure 2B). From 2,466 peptides presently identified, 6 peptides intersect with *Magalhães* et al.*,*^6^ 76 peptide with *Parker* et al.*,*^22^ while 42 peptides were common to all these three mentioned studies (Figure 2B; Table S6).

**Figure 3:**
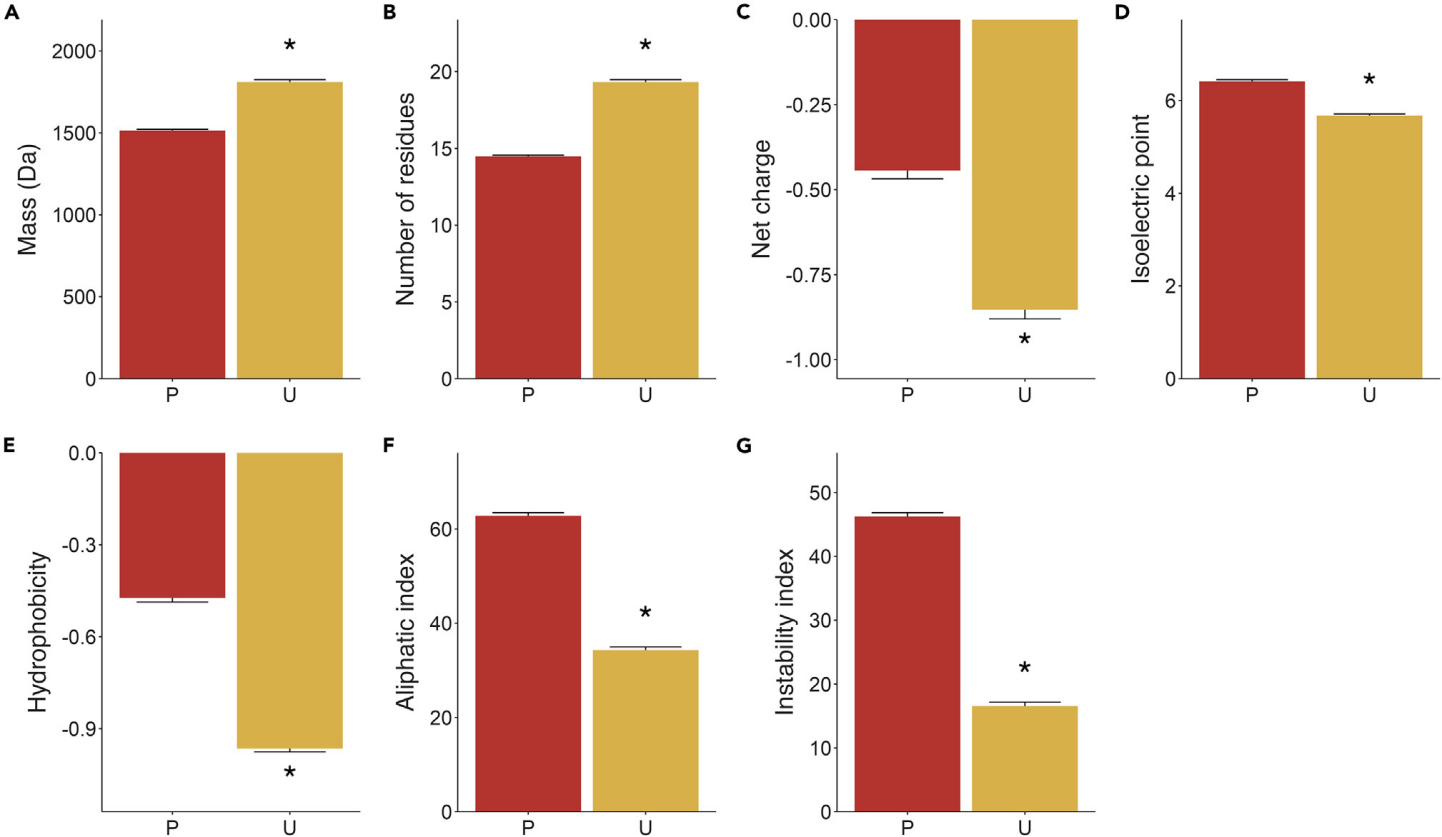
Biochemical comparisons between plasma and urine peptides Identified plasma peptides (P) were both herein and previously by Magalhães et al.^5^ and *sC*, Parker et al.,^25^ and urine peptides (U) from previously reports by Magalhães et al.^5^ and Wendt et al.^3^ (A) Peptide mass. (B) Number of residues. (C) Net charge. (D) Isoelectric point. (E) Hydrophobicity. (F) Aliphatic index. (G) Instability index. ∗p ≤ 0.05 vs. P.

**Figure 4:**
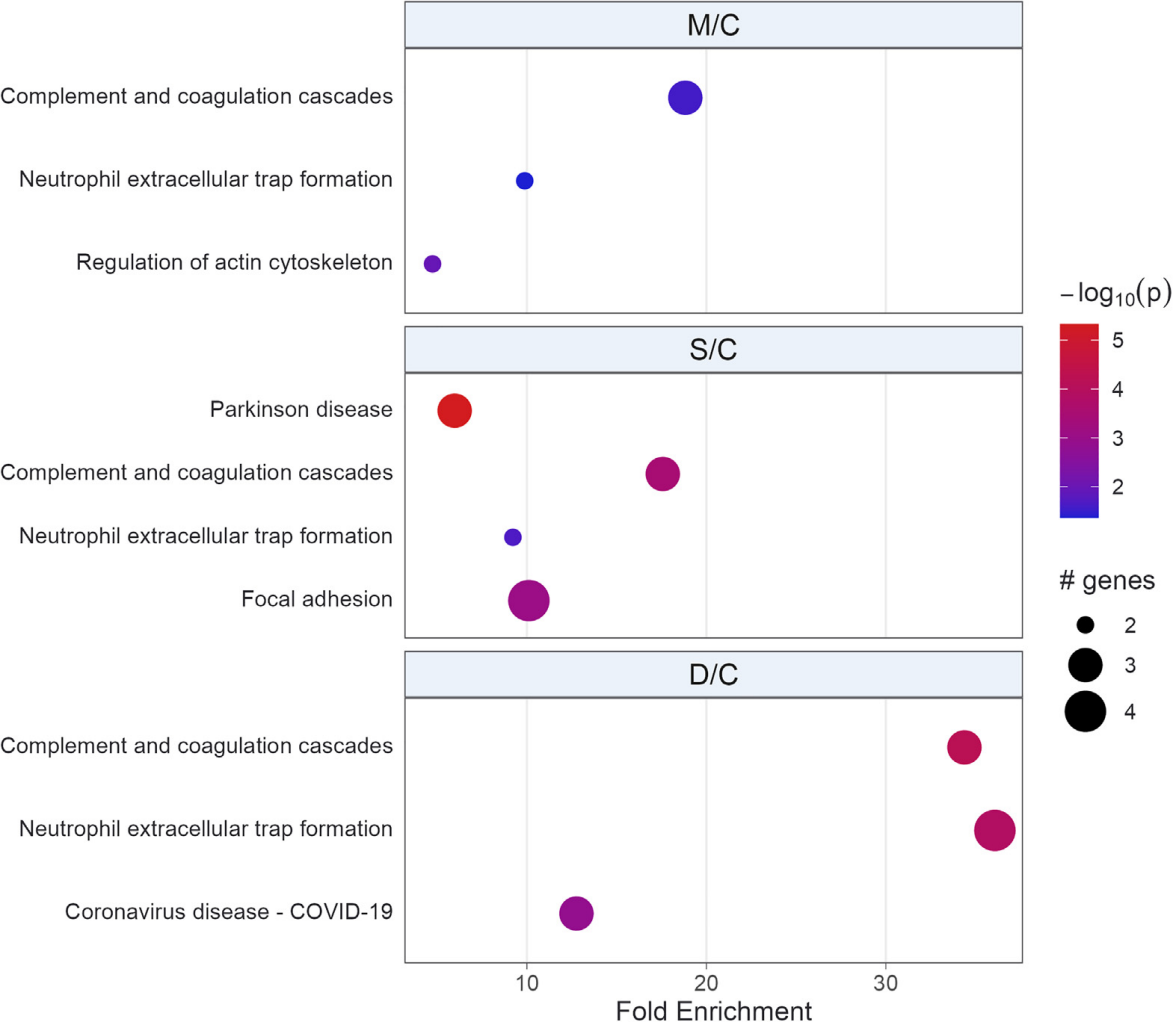
PathfindR bubble plot of Biogrid PIN represented by Kyoto Encyclopedia of Genes and Genomes (KEGG) terms Enriched PIN were identified using genes encoding the proteins that originated the differentially expressed peptides. The x axis corresponds to fold enrichment values, while the y axis indicates the enriched pathways with at least two genes. Bubble size indicates number of differentially expressed genes (DEGs) in the given pathway. Color indicates −log_10_(p value) value; the more it shifts from purple to red, the more significant the PIN was enriched.

**Figure 5:**
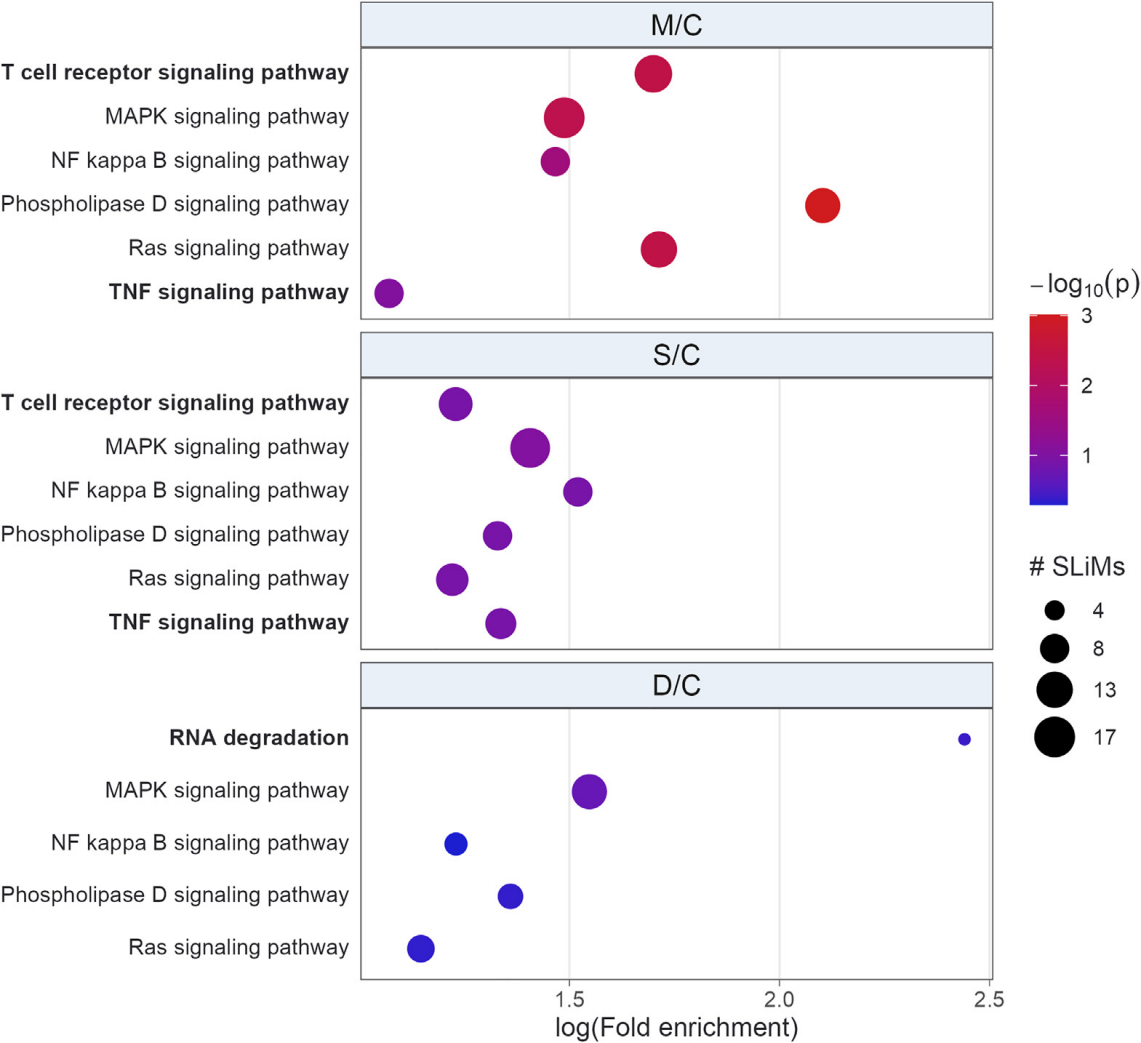
Bubble plot of overrepresented differentially regulated peptides docking or SLiMs KEGG terms On Y axis are terms with p value ≤ -log10(p value) and part of top three lowest p values of at least one group comparison (M/C, S/C or D/C). Bold terms were enriched on groups M/C and S/C, or exclusively on group D/C. Bubble size represents number of SLiMs related to a given term. Color scale indicates -log10(p value); redder the color, more significantly pathway is enriched.

**Figure 6:**
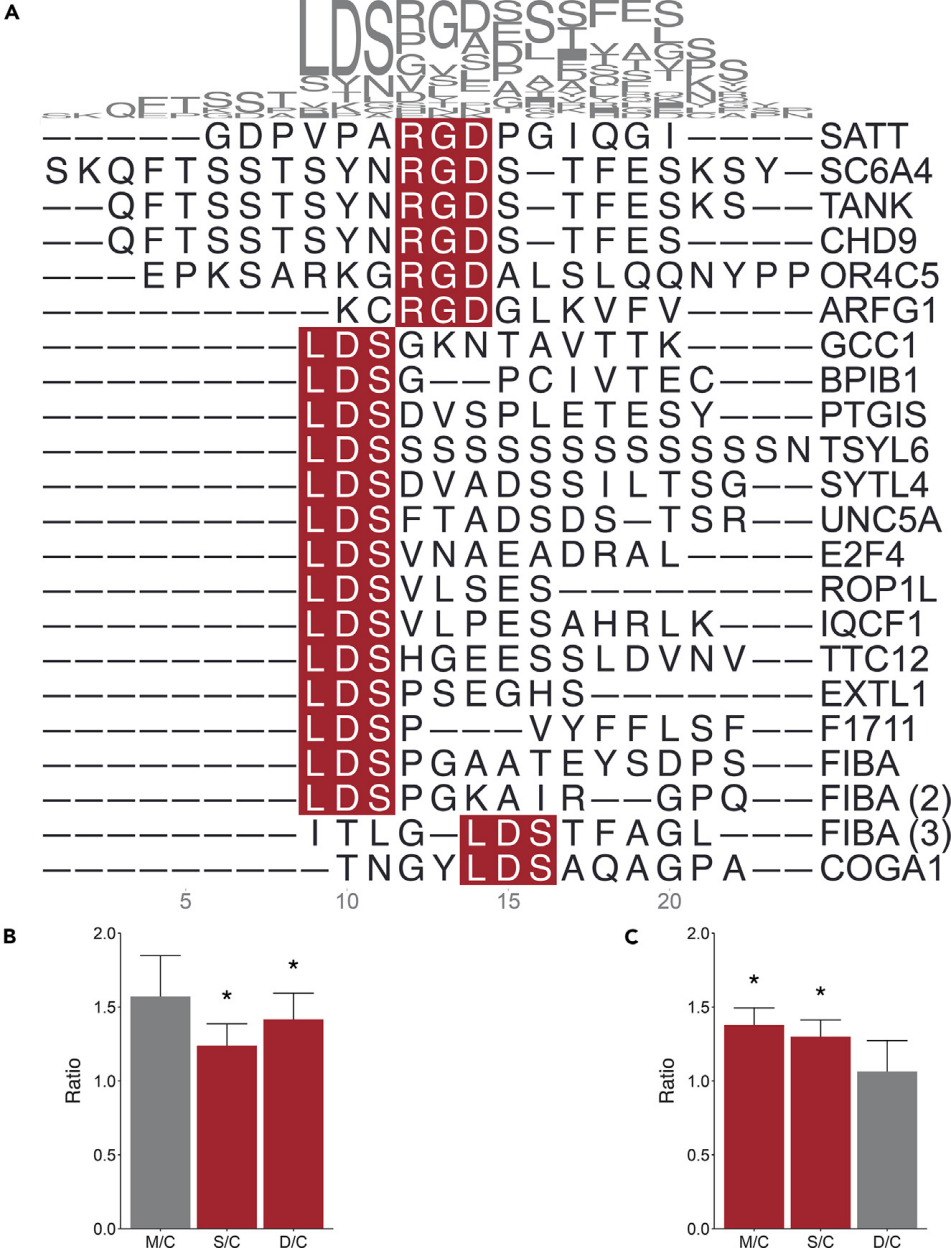
Multiple sequence alignment of plasma peptides identified herein that contain the RGD or LDS SLiMs (A) Peptides containing either the motif RGD, (B) or LDS, (C) and their respective relative ratios in M/C, S/C, or D/C. For better visualization, extreme peptides ratios values used to produce the plot were capped at 10 and 0.5. ∗Peptide ratio significantly greater than one (p ≤ 0.05). The peptide-precursor protein was shown after each peptide sequence. Proteins which generated more than one peptide were identified with unique ID in parentheses.

The entire pool of plasma peptides identified in this study and previously,^6^^,^^22^ was compared to the collection of previously reported urinary peptides.^4^^,^^6^ These data suggested that plasma peptides, compared to urinary peptides, were: (1) significantly lighter (Figures 3A and 3B), (2) shorter (Figures 3B and 3C), (3) less negatively net charged (Figures 3C and 3D), (4) of higher isoelectric point (Figures 3D and E), (5) less hydrophilic (Figures 3E and F), (6) of greater aliphatic index (Figures 3F and G), and (7) of greater instability index (Figure 3G). Thus, these data suggest the existence of a distinctive large pool of plasma peptides, whose biological significance have not yet been investigated.

Protein-protein interactions (PPI) networks (PIN) were constructed using genes encoding the proteins that originated the differentially expressed peptides identified from groups M/C, S/C, and D/C. These analyses sheds light on the biological significance of differentially regulated peptides, revealing that their enriched terms were commonly associated with SARS-CoV-2 infection (Figure 4). A higher fold enrichment in D/C group compared to M/C or S/C groups was identified for term related to complement and coagulation cascades, and neutrophil extracellular trap formation. Parkinson’s disease term was enriched exclusively in S/C group (Figure 4), which could indicate propensity of these patients to develop post-COVID-19 neurological symptoms. The coronavirus disease/COVID-19 term was enriched only in the D/C group (Figure 4).

Ligand short linear motifs (SLiMs) are functional modules that participate in PPI without requiring stable tertiary structure to accomplish their function.^26^ Thus, over-representation analysis (ORA) using SLiMs in differentially regulated peptides evince their possible role in interfering with PPI, and impacting the severity of SARS-CoV-2 infection (Figure 5). The prediction tool from ELM, a dedicated database and exploratory server for over 300 SLiMs classes, with experimental evidence manually curated from over 3,800 scientific publications,^26^ was employed for these analyses.

The ELM tool predicted an average of 5.31 SLiMs per peptide, among 108 (94%) out of 114 (100%) distinct differentially regulated peptides from M/C, S/C, or D/C groups. After filtering the SLiMs to remove duplicates and to keep only those with annotated instances of *Homo sapiens* from the classes docking or ligand, a significant overrepresentation of the MAPK, NF-κB (NF-kB), phospholipase D, and Ras signaling pathways were identified in all group comparisons. However, T cell receptor and tumor necrosis factor (TNF) signaling pathways were enriched only in the M/C and S/C groups, while RNA degradation was exclusively enriched in D/C.

Using SLiMs, as a framework to conduct ORA can offer insights into the processes taking place in the body. Indeed, integrins possess the capability to bind to a vast array of ligands harboring SLiMs such as an RGD,^27^ LDS or LDI^28^; the presence of those cells’ attachment motifs in spike (S) protein of SARS-CoV-2 (S-SARS-CoV-2) indicates its usage by the virus’s cell entry system for pathogenic hijacking.^27^ RGD and LDS, but not LDI, peptides were identified multiple times in this study (Figure 6A); similarly, peptides containing the RGD and LDS motifs were also identified in previous plasma peptidome studies.^6^^,^^22^ The ratio of peptides containing either RGD or LDS motif was not differentially regulated within any group (data not shown). However, considered as a class of peptides, those containing the RGD motif were increased in S/C and D/C groups, but not in the M/C group (Figure 6B). Peptides containing the LDS motif were increased in M/C and S/C groups, but not in D/C group (Figure 6C).

## Discussion

One of the major findings of the present report was to reveal in human plasma the presence of a large pool of peptides derived from intracellular protein precursors (i.e., InPeps). The relative levels of InPeps frequently identified in SARS-CoV-2-infected patients was increased, suggesting these to be interesting candidates for disrupting the PIN related to SARS-CoV-2 infection severity. The biological function of the InPeps identified herein remains elusive and deserves further investigation, as prior evidences indicates the potential for both biological^29^^,^^30^^,^^31^^,^^32^ and medical^4^^,^^16^^,^^17^^,^^18^^,^^19^^,^^20^^,^^21^^,^^25^ relevance of plasma-resident peptides.^6^^,^^22^^,^^33^

InPeps IKERVPDSPSPAPSLEE and KERVPDSPSPAPSLEE, derived from the intracellular protein DACH1, were identified in patients with a frequency of 58% and 33%, respectively; these peptides were significantly increased in SARS-CoV-2-infected patients (i.e., M/C, S/C and D/C). A similar DACH1-derived peptide lacking three N-terminal residues, VPDSPSPAPSLEE, identified with a frequency of 66.67% among all patients, was also increased in SARS-CoV-2-infected patients with moderate or severe symptoms. These data imply that proteolytic processing (i.e., by aminopeptidases) differentially regulates the levels of plasma peptides, according to the severity of COVID-19. It is worth mentioning that InPeps functions correlates with those of the precursor proteins,^34^^,^^35^^,^^36^^,^^37^ indicating that InPeps could be contributing to SARS-CoV-2 symptoms playing a role similar to those of their precursor proteins. In line with that, a recent report showed that the protein DACH1 is associated with SARS-Cov-2 infection^38^; in addition, the expression of DACH1 is relatively increased in patients infected with influenza.^39^ DACH1 represses the transcriptional level of matrix metalloprotease 9 (MMP9) by interacting with p65 and c-Jun at the NF-κB and AP-1 binding sites in the MMP9 promoter, respectively.^40^ MMP9 was also suggested as an early indicator of respiratory failure in patients infected by SARS-Cov-2.^41^^,^^42^ The association of DACH1 and p65 promotes the recruitment of HDAC1 to the NF-κB binding at the MMP9 promoter, reducing p65 acetylation level and transcriptional activity, and inhibiting the metastasis of breast cancer cells downregulating the expression of MMP9,^40^ and reducing the canonical NF-κB-driven inflammation.^43^

Plasma peptides reported herein and elsewhere^6^^,^^22^ have very little overlap to previously reported urinary peptides.^4^^,^^6^ This fact may indicate that renal clearance selects specific peptides for excretion based on their biochemical characteristics; thereby, enabling the persistence of numerous peptides in the plasma (i.e., human plasma resident peptides). This observation suggests demonstrate a potential undiscovered pivotal role for plasma peptides in human physiology, as well as in disease states as SARS-CoV-2 infection. These distinguishable biochemical characteristics of plasma and urine peptides shown in the present study, provides a rationale for improving the pharmacokinetics of functional rationally design peptides. This could be of interest to improve the half-life of peptides in the plasma, reducing rapid urinary excretion.

The protein from which the peptide originates can provide valuable information about its biological role.^34^^,^^35^^,^^36^^,^^37^ Supporting this notion, PIN enrichment analyses revealed coronavirus disease/COVID-19 enrichment in D/C group only. Furthermore, these analyses showed a higher fold enrichment of the PIN terms related to complement and coagulation cascades, and neutrophil extracellular trap formation in patients that died from SARS-CoV-2 infection. Additionally, considering the frequent association of neurological disorders such as Parkinson’s disease with post-COVID-19 sequelae,^44^^,^^45^^,^^46^ the enrichment of this term exclusively in the S/C group may suggest that patients with a severe SARS-CoV-2 infection are more prone to developing Parkinson’s-like symptoms.

SLiMs are compelling candidates for deciphering the roles played by peptide sequences, suggesting that the biological functions of peptides can be predicted from their SLiMs.^47^^,^^48^ Specifically, these analyses can reveal enriched terms commonly associated with SARS-CoV-2 infection, underscoring the potential usefulness of SLiMs as a means for comprehending the pathophysiology of this disease. Such functional motifs consist of short linear sequences, typically containing 3–15 residues,^49^^,^^50^ and can serve as a proxy for peptides’ biological activities. In fact, an in-depth exploration through ORA of differentially regulated peptides’ SLiMs brought up a large number of enriched terms commonly linked to SARS-CoV-2 infection.^51^^,^^52^ To spotlight the relationship between findings from SLiMs ORA and SARS-CoV-2 infection, it is noteworthy that in all infected groups, NF-kB, a central player in SARS-CoV-2 cytokine storm,^53^ is enriched alongside the MAPK signaling pathway. In circulating immune cells, MAPK is activated during the active phase of SARS-CoV-2 infection, especially in severe cases.^54^ Additionally, activation of this pathway could partially be responsible for the increase in platelet activation and aggregation observed with this infection.^55^ Considering that Ras serves as the initial module in the MAPK cascade, it is unsurprising to observe its enrichment across all infected groups. Significance of Ras in COVID-19 becomes apparent through evidence showcasing that inhibition of its downstream axis impedes SARS-CoV-2 replication.^56^ Another aspect of SLiMs ORA is what distinguishes a fatal from a non-fatal SARS-CoV-2 infection. Viral replication can be potently inhibited through selective activation of the TLR3/TLR4-IRF3 pathway.^57^ SARS-CoV-2 infected patients with an unfavorable outcome presented lower TLR3 expression and enhanced expression of TLR4, which could be related to the inflammatory response of patients with severe COVID-19.^58^ Enrichment of T cell receptor and TNF signaling exclusively in patients who survived suggests that peptides, through their SLiMs, competitively inhibit protein interactions^59^^,^^60^ that would otherwise trigger extensive T cell activation and TNF signaling, which are associated with poor clinical outcomes.^61^^,^^62^ Moreover, exclusive enrichment of RNA degradation in individuals who succumbed to the infection can indicate pathogen’s exploitation of cellular machinery to accelerate degradation of cytosolic cellular mRNAs, which facilitates viral takeover of the mRNA pool in infected cells.^63^^,^^64^

The S-SARS-CoV-2, which plays a key role in the receptor recognition binding and cell membrane fusion process, is composed of subunits S1 and S2. The S1 subunit of S-SARS-CoV-2 contains a receptor-binding domain (RBD) that recognizes and binds to the host receptor angiotensin-converting enzyme 2 (ACE2); the S2 subunit of S-SARS-CoV-2 mediates viral cell membrane fusion.^65^ The mechanisms surrounding SARS-CoV-2 infection have been widely attributed to ACE2-mediated pathways.^66^^,^^67^ However, SARS-CoV-2 infection was observed in many extra-pulmonary tissues, such as brain tissue, cardiovascular tissue, and lymphoid tissue, where ACE2 expression was very low.^68^ Integrins are a family of α/β heterodimeric cell surface adhesion receptors, which have also been suggested as a possible receptor candidate for SARS-CoV-2 cell infection.^28^^,^^65^^,^^69^ The RBD of S-SARS-CoV-2 has three potential integrin-binding motifs: RGD (Arg403-Gly404-Asp405), LDS (Leu441-Asp442-Ser443) and LDI (Leu585-Asp586-Ile587).^28^^,^^65^^,^^69^ The S-SARS-CoV-2 was shown to depend on its integrin RGD motif to elicit vascular leakage events, which can be prevented by the RGD-cyclic peptide compound cilengitide.^70^^,^^71^ Herein, the relative ratio of InPeps containing RGD or LDS motifs were reported to be increased in patients infected by SARS-CoV-2. Moreover, peptides presenting LDS motif were increased in M/C and S/C groups, but not in D/C group, which may help patients to survive the SARS-CoV-2 infection. On the other hand, binding of plasma RGD peptides to integrins may prevent the binding of S-SARS-CoV-2, thereby leaving it unhindered to interact with ACE2 facilitating a more robust viral cell entry, as previously suggested.^70^ Plasma peptides containing RGD motifs, might also regulate the activation of transforming growth factor β,^72^ which could lead to tissue fibrosis and augmented coagulation.^73^ Therefore, the relative increase in peptides containing the RGD motif in patients from D/C groups may contribute to SARS-CoV-2 fatal effect. Nonetheless, these captivating possibilities warrant further experimental exploration to unveil additional clinical implications of plasma peptides/InPeps.

### Limitations of the study

The present study has some important limitations. This was a single-center study. Blood samples from SARS-CoV-2-infected patients from groups M, S, and D were collected early during the first wave of infection. Blood from SARS-CoV-2 non-infected individuals was collected later during the course of the COVID-19 pandemic, with unusual limited clinical information about the patients. There were no available data about the vaccinal status or previous SARS-CoV-2 infections from control individuals non-infected by SARS-CoV-2 at the moment of the blood test. Therefore, differences in the relative ratio of human plasma peptides presented herein should be taken as the differences between individuals that: (1) tested positive for SARS-CoV-2 infection by RT-PCR test in nasopharyngeal and throat swabs, and by typical chest computed tomography (CT)-scan findings at the moment of their blood collection; and (2) compared to individuals that tested negative for SARS-CoV-2 infection by RT-PCR test in nasopharyngeal and throat swabs at the moment of their blood collection. Therefore, SARS-CoV-2 derived peptides were identified in blood of individuals that tested either negative or positive for SARS-CoV-2 infection at the moment of blood collection (data not shown). Differences in peptidome caused by distinctive SARS-CoV-2 variants or those caused by vaccination status were beyond the scope of this study. The statistical analyses presented herein have not been further correlated with additional clinical conditions of the patients/individuals, including obesity, diabetes, and/or hypertension, hospitalization period (days), need for mechanical ventilation, or time under mechanical ventilation if needed (days). The limited number of differentially modulated peptides according to sex suggests the need for a larger patient cohort to further investigate the modulation of plasma peptidome. Urine and plasma were not simultaneously collected from patients investigated in the present study, which prevented the parallel analysis of plasma and urine peptidome under similar experimental protocols.

### Conclusions

SARS-CoV-2 infection perturbs the plasma peptidome. PIN related to SARS-CoV-2 infection was deciphered through peptidome signature. The presence of resident InPeps in human plasma may have implications for health and diseases, and differs from the urinary profile indicating selective renal filtration.

## STAR★Methods

### Key resources table

**Table:** 

REAGENT or RESOURCE	SOURCE	IDENTIFIER
**Biological samples**		

Human plasma	Emergency Service - HCFMUSP	N/A

rowhead**Deposited data**		

Mass spectrometry data	This paper	http://www.ebi.ac.uk/pride/archive/projects/PXD040580 ftp://ftp.pride.ebi.ac.uk/pride/data/archive/2023/04/PXD040580.

**Software and algorithms**		

Mascot Daemon (v.2.3; search engine v.2.8)	Matrix Science	https://matrixscience.com
R (v.4.1)	R Core team	
R library pathfindR (v.1.6.4)	Ferro, E.S.et al.^32^	https://cran.r-project.org/web/packages/pathfindR/index.html
R library MSnbase (v.2.24.2)	Huang, M.et al.^28^	https://bioconductor.org/packages/release/bioc/html/MSnbase.html
ELM prediction tool	Gewehr, M.C.et al.^25^	http://elm.eu.org/search.html
ClustalW	de Araujo, C.B.et al.^34^	http://clustal.org

### Resource availability

#### Lead contact

Further information and requests for resources and reagents should be directed to and will be fulfilled by the lead contact, Emer S. Ferro (eferro@usp.br).

#### Materials availability

This study did not generate new unique reagents.

### Experimental model and study participant details

#### Ethical approval and informed consent

The study protocol was approved by the Research Ethics Committee (REC) of Hospital das Clínicas da Faculdade de Medicina da Universidade de São Paulo, SP, Brazil (HCFMUSP; protocol number CAAE 30417520.0.0000.0068), with written informed consent or verbal authorization documented in the patient’s charts. Patient anonymity was preserved. Written informed consent was not possible, for example, if the patient was unconscious or in acute respiratory failure. When written informed consent from the patient was not possible, informed verbal authorization from the patient or family members in the presence of witnesses was authorized by the REC. All patients were treated according to hospital protocols, which included prescription of antibiotics at admission in all cases. The study was registered in the Brazilian registry of clinical trials RBR-5d4dj5.

#### Patient admission and classification criteria

Participants were enrolled at the Emergency Service in Internal Medicine from HCFMUSP. Patients admitted to the Emergency Service with severe SARS-CoV-2 infection on admission, according to WHO guidelines,^23^^,^^24^ were enrolled. SARS-CoV-2 infection was diagnosed by a positive SARS-CoV-2 reverse transcriptase polymerase chain reaction (RT-PCR) test, employing nasopharyngeal swabs, and by typical chest computed tomography (CT)-scan findings. Individuals diagnosed by a negative SARS-CoV-2 RT-PCR test in nasopharyngeal swabs, were considered non-infected (control) individuals. All enrolled SARS-CoV-2 positive patients had hypoxemia (as defined by peripheral oxygen saturation of less than 92%) and required supplemental oxygen. Patients under 18 years old, pregnant women, and patients in end-of-life protocols were not included.

### Method details

#### Sample handling and peptide identification

Plasma was separated by centrifugation from blood collected from the brachial vein, in tubes containing anticoagulant (sodium citrate 0.109 M (3.2%) previously buffered with N-2-Hydroxyethylpiperazine-N'-2'-Ethanesulfonic acid; Hepes salt), and aliquoted in 0.5 mL samples which were kept at -80°C until use; one of these plasma samples was devoted for peptide extraction. Plasma samples (300 μL) were transferred to a protein low-binding tube, and an equal volume of 0.5% bovine serum albumin, phosphate buffered saline containing 0.05% Tween 20, pH 7.2 (sample buffer) plus two volumes of acetonitrile (to achieve 66% of acetonitrile to the final sample extraction volume) were added to the tubes. After vortexing, samples were incubated at room temperature for 60 minutes (min) and centrifuged at 12,000 x g for 5 min at 4°C. The supernatant was transferred to a clean protein low-binding tube, and the volume was reduced in a speed vacuum centrifuge (Eppendorf, Hamburg, Germany) for 3 hours (h) at 30°C. The semi-dried pellet was resuspended in 1.5 mL of ultrapure water, acidified to pH 2 with 0.1 M HCl (final concentration of 10 mM; Millipore, Burlington, MA, USA) and transferred to Amicon Ultra-4 Centrifugal Units of 10,000 Da cutoff (Millipore, Burlington, MA, USA). After centrifugation at 1,500 x g, at 4°C, for approximately 1 h, the pH of the flow through (containing peptides of molecular mass < 10,000 Da) was adjusted to 2-4 with 50% formic acid (Fisher Scientific, Pittsburgh, PA, USA). This flow-through material-containing peptides was passed through Oasis HLB 1cc (30 mg) Extraction Cartridges (Waters, Etten-Leur, NB, NL) previously equilibrated with 100% acetonitrile/0.10% formic acid (Fisher Scientific, Pittsburgh, PA, USA), and then with 5% acetonitrile/0.10% formic acid in ultrapure water. After washing with 5% acetonitrile/0.10% formic acid, peptides were eluted in 100% acetonitrile/0.15% formic acid, collected in Fisherbrand™ low-retention microcentrifuge tubes (Waltham, MA, USA), and dried in a speed vacuum centrifuge (Eppendorf, Hamburg, Germany). A peptide aliquot was resuspended in ultrapure water (100 μL) and quantified by 214 nm absorbance (NanoDrop™, Thermo Fisher Scientific, São Paulo, Brazil), using a peptide mix of known composition and concentration as the standard reference for determining peptide concentration (standard curve).^76^

Peptide samples were labeled using dimethyl isotopic labeling, as previously described^76^ (Table S1). The labeling method employed is based on the dimethylation of amine and formaldehyde groups in the presence of cyanoborohydride. The combination of regular, deuteride and ^13^C formaldehyde with regular and deuteride cyanoborohydride allowed us to use four isotopic forms as the reactions added 28, 30, 32 or 36 Da to the final mass of peptides at each available (lysine or N-terminal) labeling site, which can be observed in the MS spectrum.^76^ Briefly, 10 μg of purified peptide extract was diluted in 100 μL of triethylammonium bicarbonate (TEAB) buffer (Sigma-Aldrich, St. Louis, USA) to a final concentration of 100 mM. In the hood, 4 μL of the different isotopic forms of the formaldehydes were added at a concentration of 4%, according to the desired labeling scheme. Then, 4 μL of 0.6 M reducing sodium cyanoborohydride (NaBH3CN) were added, and samples were incubated for 16 h at room temperature, protected from light. The reaction was then quenched with 16 μL of 1% ammonium bicarbonate. Samples were placed on ice and 8 μL of CH₂O₂ (formic acid; Sigma-Aldrich, St. Louis, USA) were added. Four differentially labeled samples were pooled, desalted using Oasis HLB 1cc (30 mg) Extraction Cartridges (Waters, Etten-Leur, NB, NL), and eluted with 100% acetonitrile containing 0.15% formic acid. The samples were dried in a vacuum centrifuge and stored at −20°C until use. The amount of 0.2 μg of peptides diluted in 5 μL of 100% acetonitrile containing 0.15% formic acid were injected for all mass spectrometry analyses, as described below. Isotopic forms (28, 30, 32 or 36 Da) were alternated to ensure that samples from patients with different clinical conditions (*i.e.,* C, M, S or D) received all different markers. Samples from SARS-CoV-2-non-infected patients (C) were eventually used more than once, allowing every run to have all four markers included. These runs and the respective patients were annotated (Tables S1 and S2).

#### Mass spectrometry analyses

Mass spectrometry peptidome analyses were performed on an electron-spray mass spectrometer coupled to nano-liquid high-performance chromatography (nLC-MS/MS) and conducted at the Mass Spectrometry Facility of FIOCRUZ PARANA, PR, Brazil. Briefly, the identification of peptides sequences was performed on an Orbitrap Fusion Lumos Spectrometer (Thermo Fisher Scientific, Bremen, Germany) through a nano-electrospray ion source. Peptide separation was performed on 360 μm OD x 150 mm a column, with 3-μm C-18 beads (Dr. Maisch, 72119 Ammerbuch-Entringen, Germany). Peptides were eluted using a linear gradient of 5-40% acetonitrile, in 0.1% formic acid, for 120 min at 250 nL/min flow. Data were acquired after the generation of multiple peptides protonated by the ESI (electrospray ionization), according to the following conditions. Runtime, 120 min, polarity positive, default charge state 2, full MS, resolution 120,000, AGC target standard, maximum IT 50 ms, scan range 300 to 1500 m/z. Dd-MS2 resolution 30,000 AGC target: standard, maximum IT 54 ms, 2 s duty cycle, isolation window 1.6 m/z, (N)CE 30. Intensity threshold for MS2 2.0e4, charge exclusion 1, and >7, peptide match preferred, exclude isotopes on, dynamic exclusion time 60.0 s. Data processing and analyses were performed *in-house* using XCalibur and the Mascot software suite (search engi, as previously described.^76^^,^^77^^,^^78^ Thus, to perform data analyses, raw data files were converted into peak list format (mgf) by Mascot Daemon (v.2.3 Matrix Science Ltd, London, UK, search engine v.2.8). Before proceeding with the search for peptides, a filtering procedure using the R library MSnbase (v.2.24.2)^79^ was used to remove peaks containing the mass of contaminants present in the Mass spectrometry Contaminants Database.^80^ This step was important because, despite all the care taken to work with samples with the highest possible quality, some contaminants, such as drug vehicles, were still identified in mass spectrometry: when the peak of one of these contaminants coincides with the expected mass time for a labeled peptide, the Mascot algorithm will consider it as the peptide; consequently, later quantitation of the ratio between the labeled peptides will be influenced.

After removing contaminants, data analyses processes continued with the search for peptides using the Mascot search engine. No cleavage site was specified and a fragment ion mass tolerance of ±0.5 Da was applied to the MS and MS/MS ions. The search parameters were no enzyme specificity; precursor mass tolerance set to ±0.5 Da; no modifications included. The identified peptides were filtered to keep only those in which the ratio between the number of peptide-matched ions and the number of peptide residues minus one were greater than 0.4 (Table S2).

### Quantification and statistical analysis

#### Peptide relative levels and statistical analyses

In isotope-labeled mass spectrometry, the peptide ratio value among groups is indicative of its increase or decrease. We assessed whether the peptide ratios in patient groups (M/C, S/C, and D/C) were significantly different from the null effect of 1 using the One-Sample Wilcoxon Signed Rank Test. All analyses were performed using R (v.4.1). p-values ≤0.05 were significantly different.

#### PIN enrichment analysis and SLiMs

Active subnetworks identification in a PIN and the subsequent enrichment analyses were performed using the R library pathfindR (v.1.6.4)^81^ with the genes coding the proteins that were precursors of differentially regulated peptides as input. Biogrid was selected as the reference PIN, and KEGG as the reference gene set. Only pathways with at least two genes involved were kept. SLiMs from differentially regulated peptides were identified using the eukaryotic linear motif (ELM) prediction tool.^26^ Only SLiMs with *Homo sapiens* annotated instances from docking or ligand classes were included for further analyses. Enrichment of SLiMs selected through this process was evaluated by calculating their enrichment ORA per KEGG term, using Fisher’s hypergeometric test.^82^ The reference set for these analyses was the ELM^26^ database of SLiMs for *Homo sapiens* KEGG terms. A p value ≤0.05 were considered significant. Odds ratio was assumed as fold enrichment.

#### Multiple sequences alignments

Alignments of peptides containing arginine–glycine–aspartate (RGD) and leucine-aspartic acid-seryne (LDS) SLiMs were performed using the msa interface for ClustalW algorithm.^83^ RGD and LDS peptide ratios were assessed using the One-Sample Wilcoxon Signed Rank Test. All analyses were performed using R (v.4.1). p-values ≤0.05 were significantly different.

## Data Availability

• Mass spectrometry peptidomics data were deposited to the ProteomeXchange Consortium^74^ via the PRIDE^75^ partner repository. All accession numbers are listed in the key resources table. • Original codes and scripts used for the analyses are publicly available as of the date of publication at GitHub: github.com/lfmartucci/InPeps_COVID19. • Any additional information required to reanalyze the data reported in this paper is available from the lead contact upon request.

## Supplemental information

Document S1. Table S5

Table S1. Labeling description, related to mass spectrometry label scheme, related to STAR Methods

Table S2. Peptide identification and semi-quantitation info, related to STAR Methods

Table S3. Patients’ clinical information, related to Table 1

Table S4. Peptides’ frequency and p-value, related to Figure 1

Table S6. Peptides’ comparative analyzes, related to Figure 2
