# Supplementary material for: Intracellular peptides in SARS-CoV-2-infected patients
Source: iScience. 2023 Aug 6;26(9):107542. doi: 10.1016/j.isci.2023.107542 (PMC10448160; doi:10.1016/j.isci.2023.107542)
Supplement: Document S1. Table S5 [file mmc1.pdf]

iScience, Volume *26*

Supplemental information

Intracellular peptides

in SARS-CoV-2-infected patients

Luiz Felipe Martucci, Rosangela A.S. Eichler, Renée N.O. Silva, Tiago J. Costa, Rita C. Tostes, Geraldo F. Busatto, Marilia C.L. Seelaender, Alberto J.S. Duarte, Heraldo P. Souza, and Emer S. Ferro

Supplemental data items

Table S5. Peptide group comparisons according to sex, related to Table 2

	M/C		S/C		D/C	
	Male	Female	Male	Female	Male	Female
APOC2						
GIFTDQVLSVLKGEE	↔	↑	↔	↑	↑	↔
ATP7A						
VESTSNSPSSS	↔	↑	↔	↑	↔	↑
DACH1						
IKERVPDSPSPAPSLEE	↑	↑	↑	↔	↔	↔
KERVPDSPSPAPSLEE	↑	↑	↑	↑	↔	↔
VPDSPSPAPSLEE	↔	↑	↔	↔	↔	↔
DACH2						
ESPSPAPSLEE	↔	↔	↔	↔	↔	↔
KERIPESPSPAPSLEE	↔	↔	↔	↑	↔	↔
FAT2						
DGSDVSK	↓	↔	↔	↔	↓	↔
FIBA						
ADSGEGDFLAEGGGVR	↔	↔	↔	↑	↔	↔
DSGEGDFLAEGGGVR	↔	↔	↔	↔	↔	↔
GEGDFLAEGGGVR	↑	↑	↔	↔	↔	↔
SGEGDFLAEGGGVR	↑	↔	↔	↔	↔	↔
IRGQ						
LLALPPASPSAARTKA	↔	↔	↔	↔	↔	↔
KNG1						
RPPGFSPFR	↔	↔	↔	↔	↔	↔
ZN541						
SPSEESPPGPGG	↔	↔	↔	↔	↔	↔

Footnote: Individuals were clinically classified as: patients infected with SARS-CoV-2 not mechanically ventilated or moderate infection (M); 2) patients infected with SARS-CoV-2 mechanically ventilated or severe infection (S); 3) patients infected with SARS-CoV-2 with severe infection that led to death (D); non-SARS-CoV-2-infected individuals (C). The relative levels of the indicated peptide were identified as follows: M/C, moderate *vs.* control; S/C, severe *vs.* control; D/C, severe *vs.* control. Symbols indicate: ↔, no changes in peptide ratio $p>0.05$; ↑↓, respectively, peptide ratio either increased or decreased, $p<0.05$.
